# Fine particulate matter-sudden death association modified by ventricular hypertrophy and inflammation: a case-crossover study

**DOI:** 10.3389/fpubh.2024.1367416

**Published:** 2024-05-21

**Authors:** Kristen M. Rappazzo, Nicole M. Egerstrom, Jianyong Wu, Alia B. Capone, Golsa Joodi, Susan Keen, Wayne E. Cascio, Ross J. Simpson

**Affiliations:** ^1^U.S. Environmental Protection Agency, Office of Research and Development, Center for Public Health and Environmental Assessment, Research Triangle Park, NC, United States; ^2^Gillings Global School of Public Health, University of North Carolina at Chapel Hill, Chapel Hill, NC, United States; ^3^Division of Environmental Health Sciences, College of Public Health, The Ohio State University, Columbus, OH, United States; ^4^Division of Cardiology, Department of Medicine, University of North Carolina at Chapel Hill, Chapel Hill, NC, United States; ^5^Department of Family Medicine, University of Maryland Medical Center, Baltimore, MD, United States; ^6^Division of Cardiology, David Geffen School of Medicine at UCLA, Los Angeles, CA, United States; ^7^Department of Cardiovascular Medicine, Heart and Vascular Institute, University of Pittsburgh Medical Center, Pittsburgh, PA, United States

**Keywords:** particulate matter, sudden death, modification, greenspace, left ventricular hypertrophy, inflammation, arrhythmia risk

## Abstract

**Background:**

Sudden death accounts for approximately 10% of deaths among working-age adults and is associated with poor air quality. Objectives: To identify high-risk groups and potential modifiers and mediators of risk, we explored previously established associations between fine particulate matter (PM_2.5_) and sudden death stratified by potential risk factors.

**Methods:**

Sudden death victims in Wake County, NC, from 1 March 2013 to 28 February 2015 were identified by screening Emergency Medical Systems reports and adjudicated (*n* = 399). Daily PM_2.5_ concentrations for Wake County from the Air Quality Data Mart were linked to event and control periods. Potential modifiers included greenspace metrics, clinical conditions, left ventricular hypertrophy (LVH), and neutrophil-to-lymphocyte ratio (NLR). Using a case-crossover design, conditional logistic regression estimated the OR (95%CI) for sudden death for a 5 μg/m^3^ increase in PM_2.5_ with a 1-day lag, adjusted for temperature and humidity, across risk factor strata.

**Results:**

Individuals having LVH or an NLR above 2.5 had PM_2.5_ associations of greater magnitude than those without [with LVH OR: 1.90 (1.04, 3.50); NLR > 2.5: 1.25 (0.89, 1.76)]. PM_2.5_ was generally less impactful for individuals living in areas with higher levels of greenspace.

**Conclusion:**

LVH and inflammation may be the final step in the causal pathway whereby poor air quality and traditional risk factors trigger arrhythmia or myocardial ischemia and sudden death. The combination of statistical evidence with clinical knowledge can inform medical providers of underlying risks for their patients generally, while our findings here may help guide interventions to mitigate the incidence of sudden death.

## 1 Introduction

Among adults aged 18–64 in the United States, non-accidental sudden deaths unrelated to previous or obvious causes account for as much as 10% of deaths and 2 million years of productive life lost (1). Estimates of sudden death vary vastly across the scientific literature, depending on the definition of sudden death used. Most definitions include timing and situational restrictions that result in the under-reporting of many cases (e.g., within 1 h of witnessed or 24 h of unwitnessed events) (2). In order to increase the likelihood of identifying missed deaths, avoid assumptions of coronary artery disease causality, and include unwitnessed deaths (3), population-level assessments seem necessary to develop more effective interventions to reduce sudden deaths.

There is an extensive body of literature causally linking ambient exposure to particulate matter less than 2.5 micrometers in aerodynamic diameter (PM_2.5_), which acts through inflammatory and oxidative mechanisms, to overall mortality, respiratory morbidity and mortality, and cardiovascular and cardiometabolic morbidity and mortality (4–19). However, there remain many uncertainties surrounding air pollution exposures and health outcomes, including the potential for different underlying etiologies for cause-specific subtypes of deaths (20–22) and the impacts of existing social and health conditions or concurrent exposures on air pollution-health associations (23–25).

**Figure 1:**
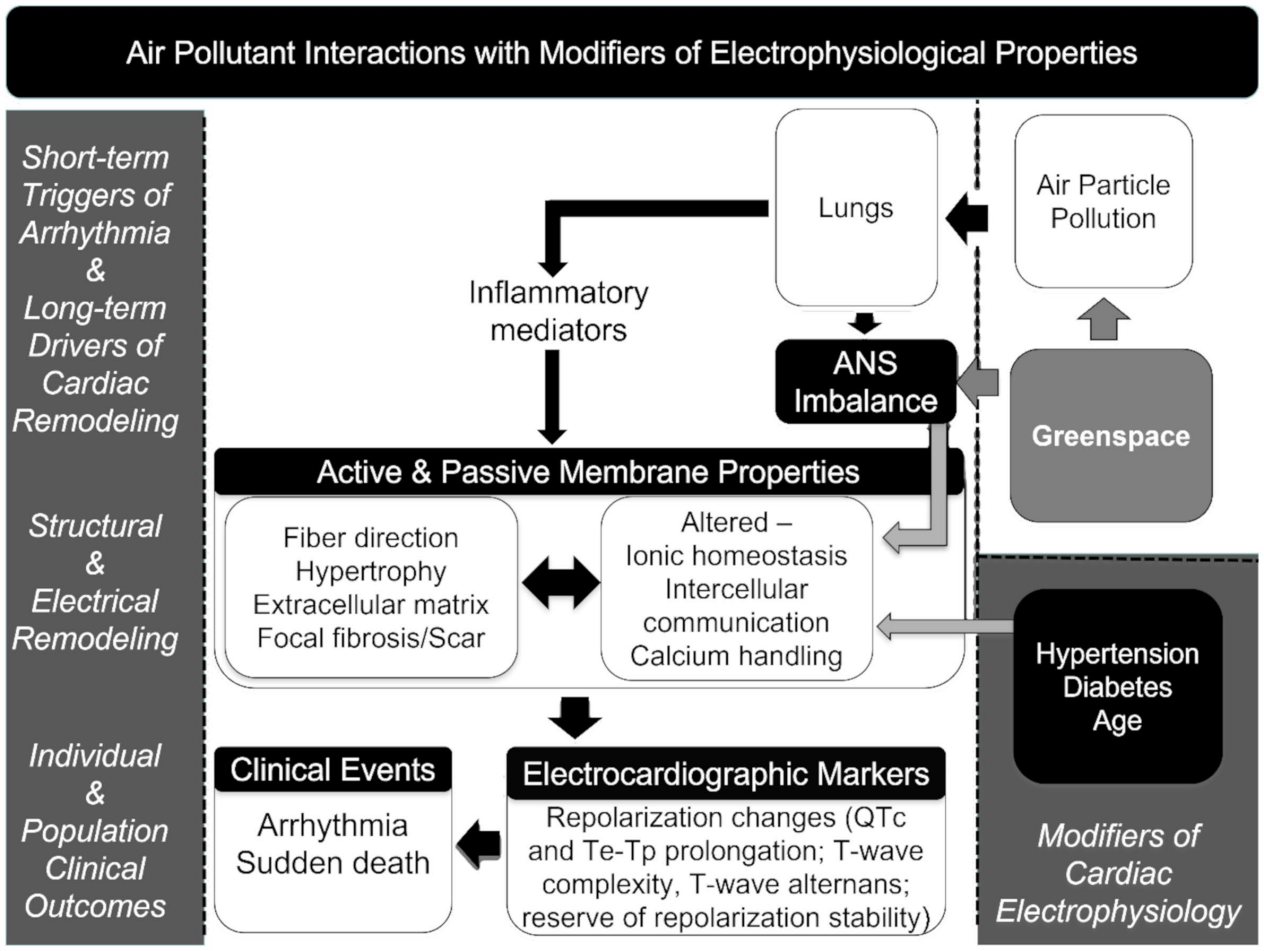
Theoretical framework connecting air pollution exposures and mortality, including electrophysiological properties (35).

We previously observed increased odds of sudden death with higher acute exposure to particulate matter less than 2.5 micrometers in aerodynamic diameter (PM_2.5_). In other analyses of this population, inflammation, left ventricular hypertrophy (LVH), stroke, chronic respiratory disease, coronary artery disease, metabolic syndrome, and lower values for greenspace metrics were associated with increased risk for sudden death (1, 26–29). Beyond our data, LVH and neutrophil-to-lymphocyte ratio (NLR) are of particular interest as they are important contributors to cardiovascular-related morbidity and mortality (30–34). Building on this information, we adapted a theoretical framework for air pollution associations with sudden death described by Cascio (35), p. 35 to highlight how the underlying conditions of individuals and their external surroundings might alter responses to ambient air pollutant exposures (Figure 1).

The goal of this analysis is to investigate potential modifiers of the PM_2.5_-sudden death association (i.e., where the response to PM varies across levels of other factors). To identify high-risk groups, we explored previously established associations between PM_2.5_ and sudden death stratified by individual and area-level factors that may modify the association between PM_2.5_ and sudden death. Defining clinical and environmental factors that either increase risk or decrease risk for sudden death might better serve to identify characteristics of an individual or a population that increase vulnerability to exposure susceptibility to a sudden death outcome. Knowledge of such clinical and environmental risks and salutary factors might improve interventional strategies to reduce sudden death in the face of an increasing likelihood of detrimental environmental exposures such as wildland fire smoke.

## 2 Methods

### 2.1 Study population and base exposure-outcome analysis

For this analysis, the study population, primary exposure, outcome, covariates, and base analysis are the same as in Rappazzo et al. (36). Briefly, data on sudden deaths in Wake County, NC, in the United States from 1 March 2013 to 28 February 2015 (*n* = 399) (1, 37, 38) was linked to PM_2.5_ concentrations, temperature, and relative humidity data acquired from the Environmental Protection Agency’s Air Quality System (39, 40). Hourly measurements of PM_2.5_, humidity, and temperature were downloaded from the single central site monitor in Wake County and averaged to daily 24-h periods (midnight to midnight) for linkage. All individuals included in this analysis experienced an out-of-hospital death that was defined as a sudden pulseless condition in the absence of terminal disease or overdose at the time of death. Emergency medical service (EMS) records were screened; following this, EMS and medical records, death certificates, medical examiner, and toxicology reports were obtained, and a panel of cardiologists adjudicated the cases to identify 399 victims of sudden death living in Wake County during this time period (26).

This study was conducted with a base population from Wake County, North Carolina. Wake County is a highly populated (approximately 1,000,000 people during the study period) and fast-growing area, with a highly educated population (53% with a bachelor’s degree compared to 32% for the United States), higher proportion of Black and African-American population (21% compared to 13% nationally), and lower poverty and disability than the United States average (7.4% vs. 11.4% poverty and 6% vs. 9% disability) (41, 42).

We used a case-crossover design with a time-stratified referent selection approach (43–47). In case-crossover designs, each individual serves as their own control, with the analytic focus on the question of when the event of interest occurs rather than whether it occurs. Control periods are in time-stratified design and are selected bi-directionally in the same calendar month-year to maximize exchangeability between event and referent periods.

Referent (control) days were selected within the same month and calendar year of the recorded death and on all the same days of the week (e.g., all Mondays within the event month/year if death occurred on a Monday). Odds ratios (ORs) and 95% confidence intervals (95%CIs) for mortality with a 5 μg/m^3^ increase in PM_2.5_ were estimated using conditional logistic regression models adjusted for temperature and relative humidity on the day of death/referent day (lag 0) and preceding lag days (lags 1 to 3) (natural cubic splines). Temperature and humidity are adjusted for as they are time-varying factors that may be related to the outcome; other factors are not adjusted for as the case-crossover approach accounts for non-time-varying factors by design. In the previous analysis, associations were elevated with exposure at a single day lag (lag 1); therefore, the analyses in this study use that as the base, unstratified, association.

### 2.2 Pathophysiologic framework and individual-level modifiers and mediators

As shown in Figure 1, the *a priori* concept driving the hypothesis tested in the study is based on a theoretical framework for air pollution associated with sudden death described by Cascio (35) in which the exposure to PM_2.5_ affects changes in inflammatory mediators and autonomic balance affecting electrophysiological properties that can be augmented in the presence of structural changes in the heart muscle (e.g., LVH) as a consequence of age and chronic hypertension increasing the risk of arrhythmia and sudden death (35). Greenspace metrics are incorporated into the framework as possible salutary factors that have the potential to reduce PM_2.5_ exposure and also modify the autonomic response to stresses.

Hypothesized individual-level modifiers and mediators investigated include those related to clinical markers of inflammation and arrhythmia risk and clinical conditions. Overweight/obesity (48) and NLR (31, 32) were chosen as markers of stress and inflammation LVH as a marker for a substrate for ventricular arrhythmia and sudden death (34). These markers were chosen due to their availability in the sudden death case registry data and their assumed place on the direct causal pathway to sudden death due to myocardial ischemia and infarction and spontaneous ventricular tachycardia and fibrillation, respectively. Specific variables were body mass index (BMI), which may be a flag for high levels of metabolic stress and inflammation related to diabetes and sleep apnea and may indicate a higher susceptibility to the impacts of air pollutants dichotomized at a BMI of less than or equal to 25 or above 25; LVH identified through review of existing echocardiograms, electrocardiographs, and autopsy reports, and classified as present in any of the three sources or absent in all, or as unknown if the subject lacked source records or if the subject had only electrocardiographs available that were negative for LVH (due to low sensitivity); and NLR dichotomized at greater than or equal to 2.15—a level that has previously been shown to be associated with increased mortality (30).

The following chronic conditions and risk factors associated with higher mortality were included in the analysis coronary artery disease, chronic respiratory disease, chronic kidney disease, diabetes, dyslipidemia, hypertension, and stroke. For analysis purposes, these conditions are stratified by the presence or absence of the condition. In addition to stratification by individual condition diagnoses, individuals were stratified by those having no clinical conditions, one clinical condition, or more than one clinical condition.

### 2.3 Area-level modifiers

We also considered greenspace and income metrics as potential area-level modifiers, as we had previously observed associations between greenspace metrics and sudden death across census tracts (28). These greenspace metrics were examined because they may promote physical activity and reduce stress, potentially act as a filter for air pollution, or act as a general buffer for environmental hazards. In this analysis, we linked the census tract greenspace metrics of greenway density, forest cover, urban land, and average tree canopy to individuals and stratified by median value across Wake County, NC. Greenspace metric details are described fully elsewhere (28, 49). Briefly, greenway density is the total length of greenways, trails, and multi-use trails in a census tract divided by the total area of that census tract. Information on greenways and trails was obtained from the GIS division of Wake County Government, North Carolina (28). Forest cover and urban land were both estimated using the National Land Cover Dataset 2011 with a 30 m spatial resolution (50). Area of forest cover (land use codes 41, 42, and 43) and urban land (land use codes 22, 23, and 24) were calculated for each census tract and divided by the total area of that census tract for a percentage metric (49). Finally, the average tree canopy was estimated using the National Land Cover Dataset 2011 Cartographic Canopy dataset (51), summing the percentage of tree canopy in each pixel across census tracts divided by the total number of pixels in the census tract. Median values across Wake County census tracts were calculated for each greenspace metric and stratified as above or below the median to signify “high” or “low” area greenspace. Area-level income has also been previously associated with sudden death (52) and was investigated here with an annual median household income at the census tract level from the 2010 Census, stratified at above or below the median for Wake County.

### 2.4 Statistical analysis

A case-crossover design cannot test non-temporal interaction effects in models. Therefore, we investigated stratified effects, in which the population is a subset to those only having or not having a particular condition, to identify potential modifiers of the base PM_2.5_-mortality association. Strata effects cannot be directly compared, so we use descriptive methods to determine differences, i.e., if stratified effect estimates appear to separate from the unstratified/base effect estimate in opposite directions, such that the stratified effect estimates would be different. For example, a base effect of 1.25 with stratified effects of 1.05 and 1.50. In addition, we performed sensitivity analyses on a population excluding individuals with chronic kidney disease and stroke (*n* = 62), as these groups were extreme in their clinical characteristics. All analyses were performed in SAS 9.4 (Cary, NC). Figures were created using R 3.5.3–4.2.3 (53) and Rstudio (54), with the tidyverse package (55).

This research was approved by the University of North Carolina at Chapel Hill’s Office of Human Research Ethics and has been approved yearly by administrative review (#14–2036). The Environmental Protection Agency’s Human Subjects Research Officer also reviewed this study and declared it non-human subjects research as all individuals were deceased at the time of data collection.

## 3 Results

### 3.1 Descriptive results

Characteristics of the study population and study area are presented in Tables 1, 2, with maps of area-level characteristics presented in Supplementary material (Supplementary Figures S1–S5). The study population is approximately two-thirds male, and one-third of Black or African-American, with a median age of 55 years. The prevalence of clinical conditions ranged from 7% (stroke) to 56% (hypertension). For the census tracts in the study area, median household income ranged from $17,000 to $169,000, with a median of $56,000 and a mean of $62,000. Greenspace metrics ranged broadly, with some census tracts having limited to no tree canopy or forest cover, whereas in other census tracks the majority of the land was classified as tree canopy or forest cover. Over the study period, PM_2.5_ ranged from 1.82 to 31.14, with a median of 10.25 and an IQR of 5.20. Seasonal distributions of PM_2.5_, relative humidity, and temperature are shown in Supplementary Table S1.

**Table 1:** Study population distributions for categorical and binary variables.

Characteristic	*N* (%)	Characteristic	*N* (%)
Coronary artery disease		Sex	
Missing	28 (7)	Missing	0 (0)
Absent	277 (69)	Female	126 (32)
Present	94 (24)	Male	273 (68)
Chronic kidney disease		Race	
Missing	28 (7)	Asian or other	10 (2)
Absent	327 (82)	Black or African-American	140 (35)
Present	44 (11)	White	249 (62)
Diabetes		BMI category	
Missing	28 (7)	Missing	0 (0)
Absent	262 (66)	<=25	181 (45)
Present	109 (27)	>25	218 (55)
Dyslipidemia		Tree canopy percentage	
Missing	28 (7)	Missing	3 (1)
Absent	225 (56)	Below census tract median	196 (49)
Present	146 (37)	Above census tract median	200 (50)
Hypertension		Greenway density	
Missing	28 (7)	Missing	3 (1)
Absent	147 (37)	Below census tract median	192 (48)
Present	224 (56)	Above census tract median	204 (51)
Left ventricular hypertrophy		Forest land cover	
Missing	96 (24)	Missing	3 (1)
Absent	241 (60)	Below census tract median	198 (50)
Present	62 (16)	Above census tract median	198 (50)
Neutrophil lymphocyte ratio		Urban land cover	
Missing	221 (55)	Missing	3 (1)
<= 2.15	55 (14)	Below census tract median	198 (50)
> 2.15	123 (31)	Above census tract median	198 (50)
Stroke		Census tract income	
Missing	28 (7)	Missing	3 (1)
Absent	345 (86)	Above median	131 (33)
Present	26 (7)	Below median	265 (66)

**Table 2:** Distributions of continuous variables across study population (age), study period (PM_2.5_), or study area (income and greenspace).

Variable	Mean	SD	Min	25th	50th	75th	Max	IQR
Age (years)	52.74	9.39	19	48	55	60	64	12
Daily average PM_2.5_ (μg/m^3^)	10.93	4.33	1.82	8.03	10.25	13.22	31.14	5.20
Area-level income ($)	61,689	24,560	17,441	46,277	56,030	72,694	169,028	26,418
Greenway density (km/km2)	0.91	0.67	0	0.49	0.72	1.16	4.05	0.67
Forest land cover (%)	22.00	16.30	0	7.97	19.05	35.37	69.49	27.41
Urban land cover (%)	29.48	19.40	1.24	12.32	28.55	41.96	95.21	29.65
Average tree canopy (%)	47.57	12.23	5.85	39.26	50.56	55.86	82.08	16.6

**Figure 2:**
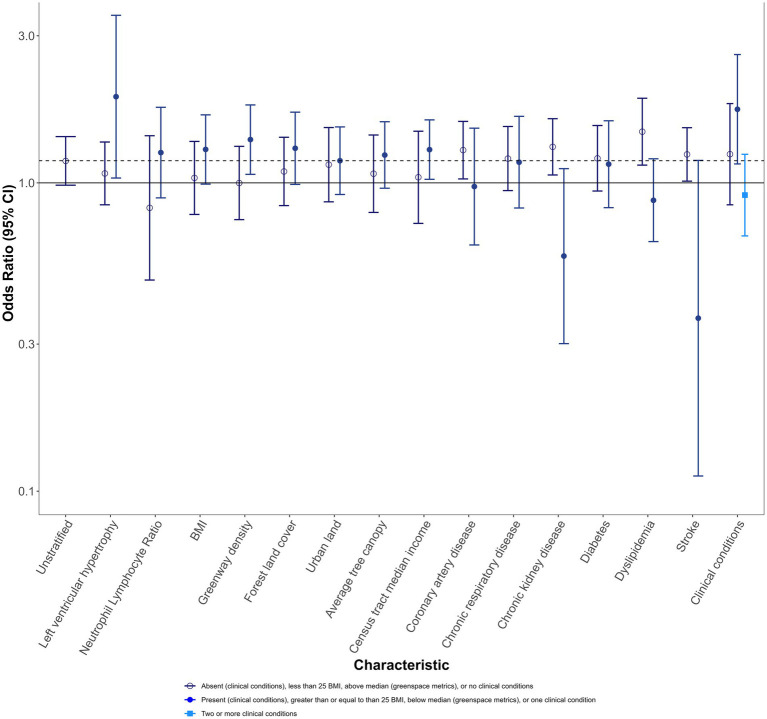
Mortality odds ratios and 95% CIs for 5 μg/m^3^ increase in PM_2.5_ 1 day before recorded sudden death, stratified by individual and area-level characteristics, for the full population. Darker blue open circle OR: absent (clinical and specific clinical conditions), less than 25 BMI, above the median (greenspace metrics), or no clinical conditions. Medium blue closed circle OR: present (clinical and specific clinical conditions), greater than or equal to 25 BMI, below the median (greenspace metrics), or one clinical condition. Light blue square OR: two or more conditions.

The unstratified estimate for a 5 μg/m^3^ increase in PM_2.5_ is 1.18 (0.98, 1.41). Stratified ORs are compared to this estimate. Results are presented for the main analysis in Figure 2; Supplementary Table S2, and for the population subset sensitivity analysis in Supplementary Table S2; Supplementary Figure S6.

### 3.2 Main analysis

Individuals with LVH had ORs elevated from the unstratified effect estimate (1.90 (1.04, 3.50)), while those without LVH had effects below the unstratified effect estimate (1.07 (0.85, 1.36)). Individuals with NLR followed a similar pattern as those with LVH, though ORs were less divergent for those with NLR. Individuals with higher BMI also had ORs elevated from the unstratified OR.

For greenspace metrics, effect estimates for those living in areas with lower greenway density, forest land cover, or average tree canopy were higher in magnitude than the overall population association. In particular, greenway density exhibited distinct separation (OR with more greenways: 1.00 (0.76, 1.31); OR with fewer greenways: 1.38 [1.07, 1.79)]. Results for census tract median income followed a similar pattern, with greater odds of sudden death for those living in below-median income areas and no evidence of effect for those living in above-median income areas.

ORs were typically higher for those without clinical conditions, with the exception of chronic respiratory disease and diabetes. When examining the number of conditions, those with no clinical conditions were similar to the unstratified effect estimate [1.24 (0.85, 1.81)], while those with a single clinical condition had an OR elevated from that effect [1.73 (1.15, 2.61)], and those with more than one clinical condition had a lower OR [0.91 (0.67, 1.24)].

### 3.3 Sensitivity analysis

Removal of individuals with stroke or kidney disease shifted stratified ORs away from the null compared to the full population, but overall patterns remained similar (Supplementary Table S2; Supplementary Figure S6). The difference between no clinical conditions and at least one clinical condition became similar to one another, suggesting that the reduction in OR observed in the main analysis was largely due to individuals with past stroke or kidney disease.

## 4 Discussion

Individuals with LVH or an abnormal NLR had a disproportionately higher likelihood of PM2.5-related death than those without these conditions, suggesting that they may be particularly susceptible to the impacts of air pollution exposures. Similarly, individuals with one clinical condition had higher PM_2.5_ ORs than the full population, though this did not hold for individuals with multiple conditions. PM_2.5_ was generally less impactful for individuals living in areas with higher levels of greenspace.

Our findings suggest that LVH and NLR may serve as clinical markers in a causal pathway whereby PM_2.5_ exposures increase the risk of sudden death. Both abnormalities may lead to cardiac repolarization changes (56) potentiating PM_2.5_ exposures that may result in ventricular fibrillation and sudden death. For example, animal evidence suggests that PM exposure may result in left ventricular remodeling (57), human evidence suggest that living near traffic may alter left ventricular mass (58, 59); long-term PM impacts these sub-clinical conditions, and that the presence of these conditions may lead to increased susceptibility to acute PM_2.5_ exposures. LVH and inflammation may be the final step in the mechanism whereby poor air quality and traditional risk factors trigger arrhythmia or myocardial ischemia and sudden death. Existing theoretical frameworks of the connection between PM_2.5_ exposure and mortality (Figure 1) may offer more insight into potential mechanisms and provide points of reference for future research and interventions (35).

### 4.1 Potential mechanisms/modes of action

Greenspace might affect PM_2.5_-mortality associations through a variety of pathways. Possibilities include exposure reduction, either due to lower air pollution emissions in those areas with higher levels of greenspace (56) or through the filtration and removal of air pollutants by vegetation (60–62). Higher levels of greenspace might also impact air pollution-related mortality by buffering through the salutary effects of stress reduction (63), improved social cohesion (64), or increased physical activity (65, 66). Greenspace metrics may also signify individuals living in wealthier areas with more opportunity to access resources, that we posit would act as an additional buffer to the negative effects of air pollution exposures.

Previous studies show that chronic conditions may increase the risk of the harmful health effects of air pollution, as they pose a more susceptible biological state; however, studies of the effects of short-term PM_2.5_ exposures on mortality in at-risk populations have produced mixed results (19). In an examination of the Nurses’ Health Study population, Puett et al. (67), observed potential differences in risk for those with and without hypertension, with all-cause mortality risks being somewhat lower in those with hypertension while fatal coronary heart disease risk was much higher in those with hypertension (67). Individuals with hypercholesteremia and higher BMIs evidenced higher risks for PM_2.5_-related all-cause mortality; but there were no differences across diabetes, median house value, or median household income strata (67). Wellenius et al. (68) found no differences in acute stroke risk with short-term PM_2.5_ exposures according to the presence of comorbid diabetes, hypertension, atrial fibrillation, or history of stroke (68). In our study, most chronic conditions appeared to confer a protective effect; this effect may have occurred due to the interaction of multiple chronic conditions or medical treatment. For example, those with cardiovascular disease and hypertension may be taking medications or behaving in ways that reduce the risk of heart attack and may counteract the detrimental effects of PM_2.5_ exposure (19, 69–71). By definition, individuals included in this study have out-of-hospital deaths, and it may be that those with these conditions are more likely to go to the hospital and experience within-hospital deaths, which would result in attenuated effects due to population selection. Alternatively, there may also be mechanistic pathways that are only observable, with specific causes of death not differentiated here.

### 4.2 Study limitations

This analysis has several potential limitations. The population is likely underpowered for full identification of potential modifiers because of the small number of subjects within each stratum. However, our sample size represents one of the largest population samples of all causes of sudden death among working-age adults. In addition, we are cautious in our interpretations of observed stratified effects and use them to identifying factors of potential mediators of air pollution on sudden death. A single site monitor was used to assign exposure and small area differences in air pollution, as between areas with and without greenspace, are not captured; though given the self-controlled design, this should not strongly impact results unless the response to PM is highly non-linear. The case-crossover design, while reducing the likelihood of unmeasured confounding, means that we cannot directly examine interaction effect estimates that would more directly identify modifying factors. Some of the potential modifiers are at the area level rather than individual level, and we cannot determine how individuals would have interacted with these, only that they lived in a census tract with those characteristics. Relatedly, we can only examine acute exposures, and there is potential for chronic air pollution to contribute to underlying conditions and susceptibility to acute air pollution exposures.

### 4.3 Study strengths

The analysis also has numerous strengths. The detailed demographic, geographic, clinical, and mortality data allowed us to explore potential modifiers of the PM_2.5_-mortality association, particularly LVH and NLR, potential direct causes of sudden death. We were also able to examine a diverse set of greenspace metrics from the National Land Cover Dataset, as the metrics analyzed may reflect different aspects of greenspace and are unavailable in the commonly used normalized difference vegetation index (28). In addition to these, other strengths include the control for confounding through study design and corresponding low expectation of residual confounding, the thorough case ascertainment that reduces the likelihood of potential selection bias, and the case definition that is expanded from the traditional definition and leads to the study populations being more racially and economically diverse than in previous studies of sudden death (36).

### 4.4 Conclusion

Here, we highlight the value of merging complex, environmental, clinical information, and geocoded events with newer, more sophisticated stratified analysis techniques. Our research approach should be generalizable to other complex clinical and research problems in cardiology, possibly to the study of the interaction of risk factors for atrial fibrillation, ischemic heart disease, heart failure, and common cardiovascular conditions with attendant morbidity, disability, and mortality. A better understanding of these complex interactions may support effective prevention efforts for targeted clinical syndromes.

Most importantly, our analysis supports the causal model of Cascio (35) that ventricular hypertrophy and inflammation are steps on a causal pathway leading from air pollution to sudden death (35). Further, our analysis supports the emerging mechanistic model that sudden death should be considered a syndrome, with causation from atherosclerosis, but, in addition, from primary arrhythmia related to structural abnormities in the myocardium, particularly ventricular hypertrophy (72). Further articulating the causal pathways leading to sudden death will help improve specificity in interventions and guide future research. We hope this research motivates more work in this field with larger cohorts. For the present, the causal pathway we outlined may serve as a guide for preliminary environmental and clinical intervention programs to mitigate sudden death. We believe our findings to be of import in light of the mounting evidence of PM as a driver of atherosclerosis progression, ischemic heart disease, heart failure, and arrhythmia. Understanding those who are most at-risk may aid in providing information on the means to limit exposures for those individuals.

## Data availability statement

The data analyzed in this study is subject to the following licenses/restrictions: Access to underlying health data may be permitted after appropriate review and approvals. Requests to access these datasets should be directed to RS, ross_simpson@med.unc.edu.

## Ethics statement

The studies involving humans were approved by University of North Carolina at Chapel Hill Institutional Review Board (#14–2036). The studies were conducted in accordance with the local legislation and institutional requirements. Written informed consent for participation was not required from the participants or the participants’ legal guardians/next of kin in accordance with the national legislation and institutional requirements.

## Author contributions

KR: Conceptualization, Data curation, Formal analysis, Investigation, Methodology, Visualization, Writing – original draft, Writing – review & editing. NE: Data curation, Writing – review & editing. JW: Data curation, Visualization, Writing – review & editing. AC: Data curation, Writing – review & editing. GJ: Data curation, Writing – review & editing. SK: Data curation, Writing – review & editing. WC: Supervision, Writing – review & editing. RS: Investigation, Project administration, Supervision, Writing – review & editing.

## Author Disclaimer

The research described in this article has been reviewed by the Center for Public Health and Environmental Assessment, US EPA, and approved for publication. Approval does not signify that the contents necessarily reflect the views and policies of the Agency, nor does the mention of trade names of commercial products constitute endorsement or recommendation for use. The Wake County EMS Data System supports, maintains, and monitors EMS service delivery, patient care, and disaster preparedness for the Wake County, NC community at large. This manuscript has been reviewed by Wake County EMS Data System investigators for scientific content and consistency of data interpretation with previous Wake County EMS Data System publications. The content is solely the responsibility of the authors and does not necessarily represent the official views of the National Institutes of Health.
