# Supplementary material for: Fine particulate matter-sudden death association modified by ventricular hypertrophy and inflammation: a case-crossover study
Source: Front Public Health. 2024 May 21;12:1367416. doi: 10.3389/fpubh.2024.1367416 (PMC11148389; doi:10.3389/fpubh.2024.1367416)
Supplement: Supplementary file 1 [file Data_Sheet_1.docx]

*Effect modification of ventricular hypertrophy and inflammation the association of fine particulate matter with sudden death: a case-crossover study in Wake County NC.*

**Supplemental materials**

Contents

[Supplemental Figure S.1: Distribution of greenway density (km/km^2^) across Wake County 2](#_Toc164167847)

[Supplemental Figure S.2: Distribution of census tract median household income (dollars) across Wake County 3](#_Toc164167848)

[Supplemental Figure S.3: Distribution of average tree canopy (percent) across Wake County 4](#_Toc164167849)

[Supplemental Figure S.4: Distribution of forest land cover (percent) across Wake County 5](#_Toc164167850)

[Supplemental Figure S.5: Distribution of urban land cover (percent) across Wake County 6](#_Toc164167851)

[Supplemental Table S.1: Seasonal distributions of PM_2.5_, relative humidity, and temperature across the study period 7](#_Toc164167852)

[Supplemental Table S.2: Mortality odds ratios and 95% CIs for 5µg/m^3^ increase in PM_2.5_ 1 day before recorded sudden death stratified by individual and area level characteristics, for full population and subset population with diagnosed kidney and stroke patients removed. 7](#_Toc164167853)

[Supplemental Figure S.6: Mortality odds ratios and 95% CIs for 5µg/m^3^ increase in PM_2.5_ 1 day before recorded sudden death stratified by individual and area level characteristics, for subset population with patients with kidney and stroke disease removed 9](#_Toc164167854)


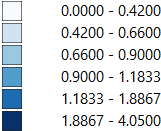
Supplemental Figure S.1: Distribution of greenway density (km/km^2^) across Wake County, six evenly spaced categories (a) and above (blue) and below median (b)
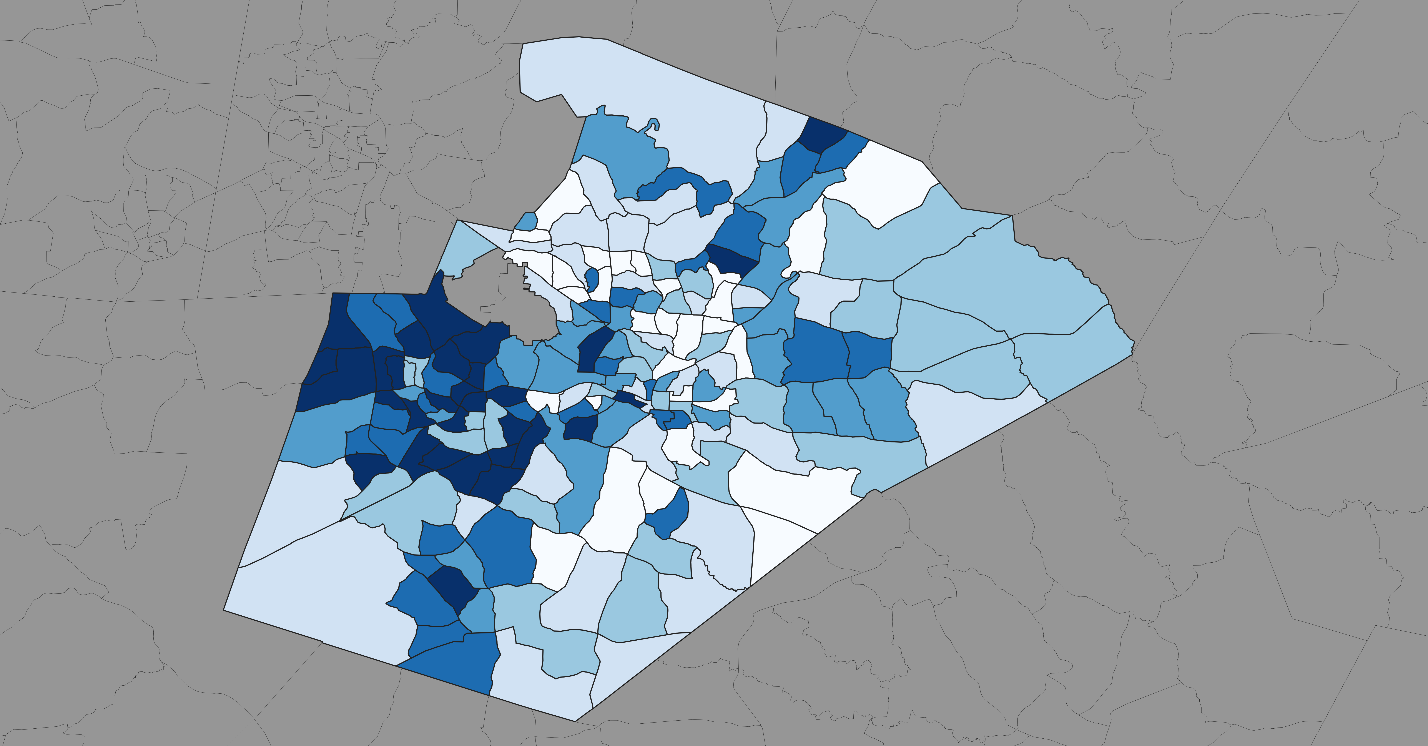


Greenway density km/km^2^

a


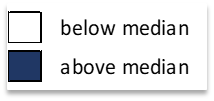

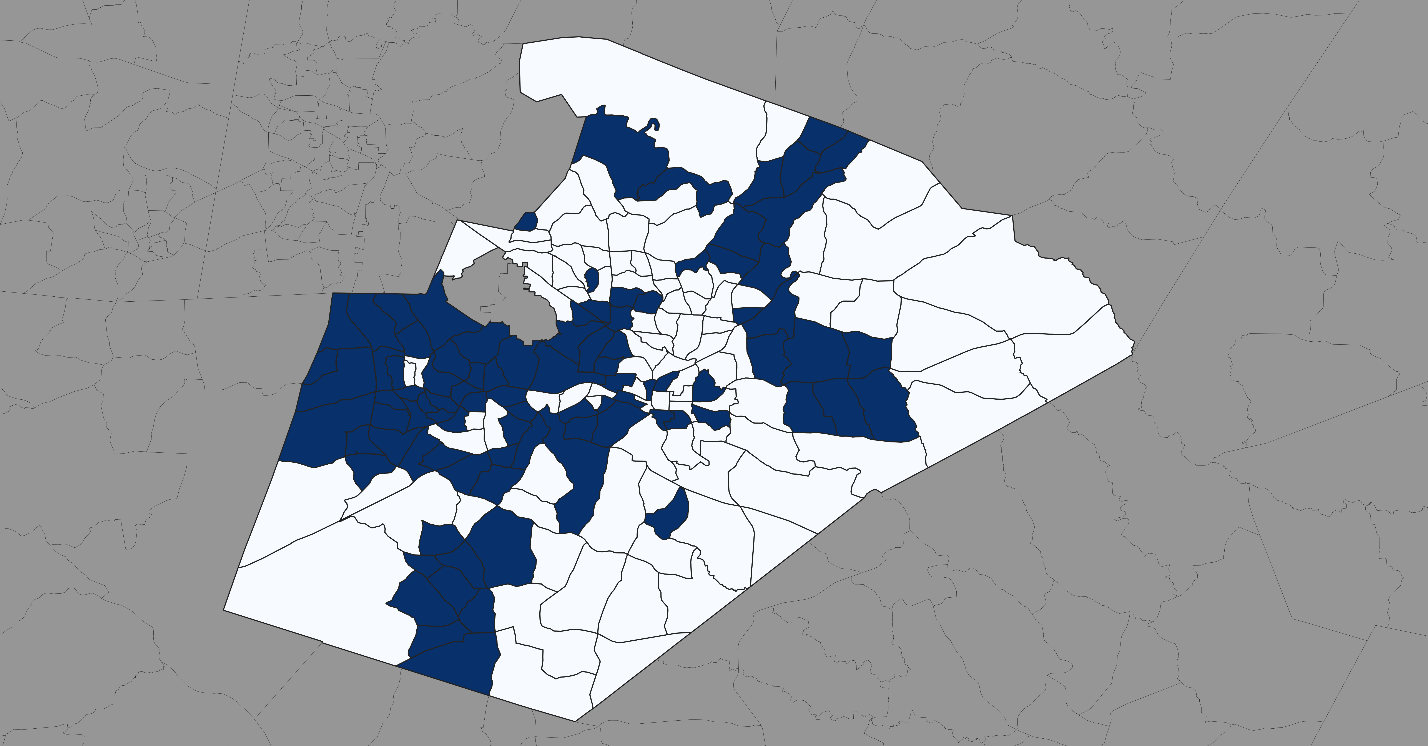


Greenway density

b


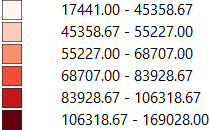
Supplemental Figure S.2: Distribution of census tract median household income (dollars) across Wake County, six evenly spaced categories (a) and above (red) and below median (b)
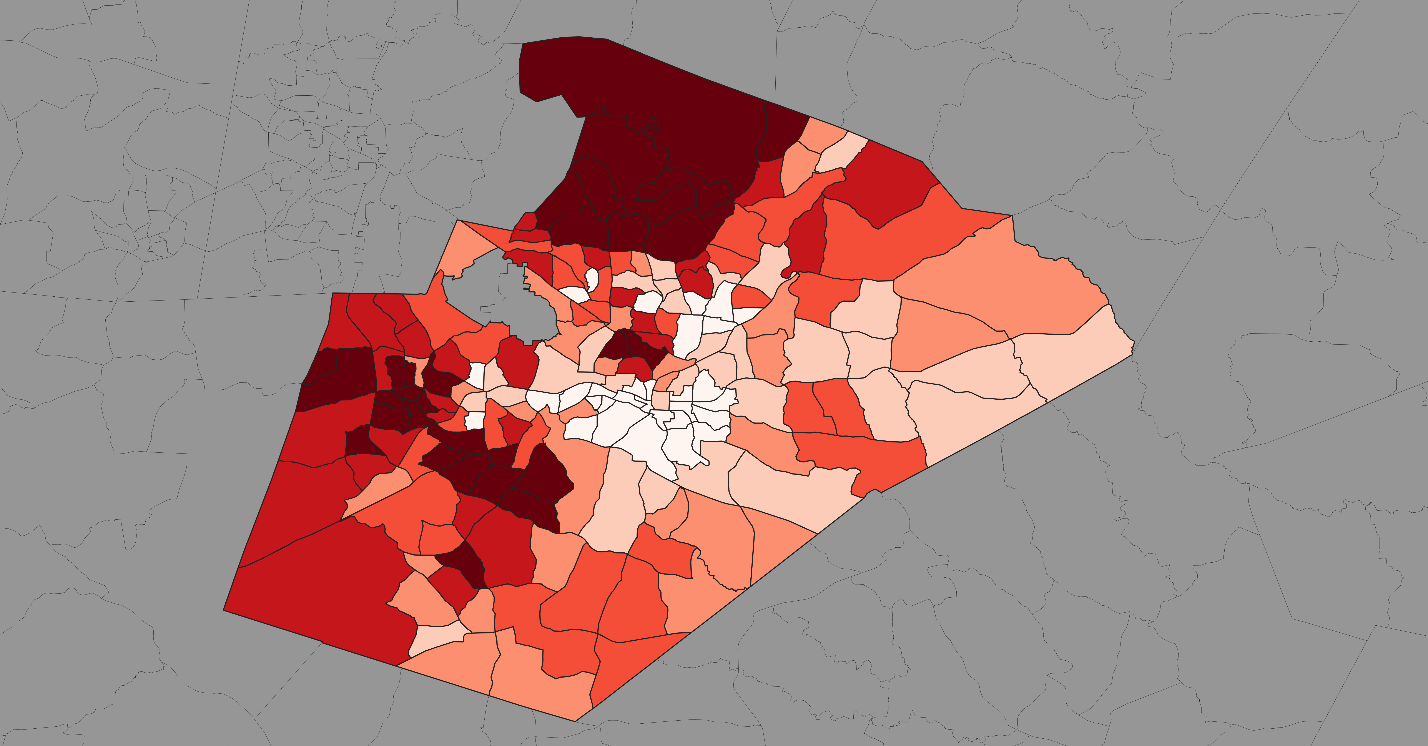


Median household income in dollars

a


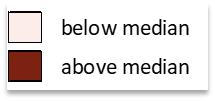

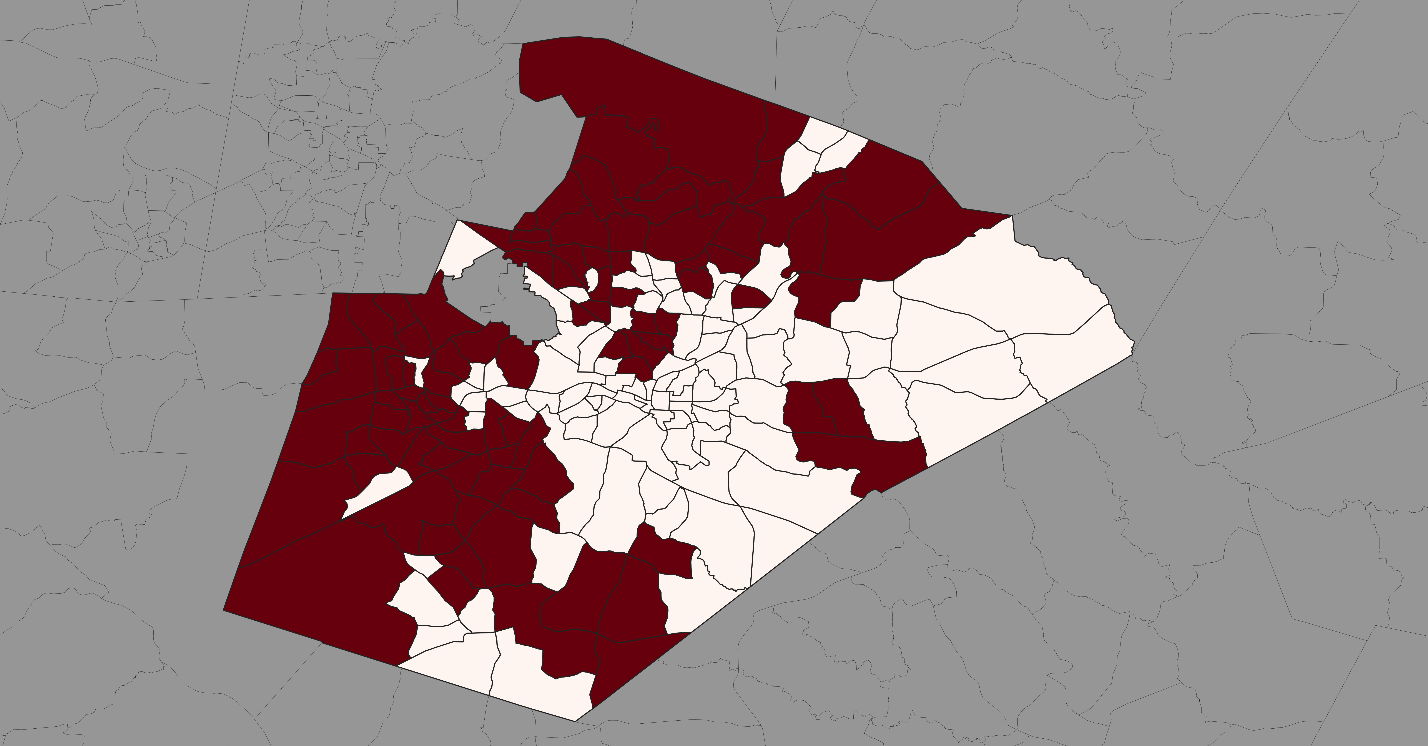


Median household income

b


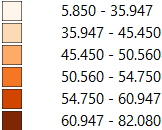
Supplemental Figure S.3: Distribution of average tree canopy (percent) across Wake County, six evenly spaced categories (a) and above (orange) and below median (b)
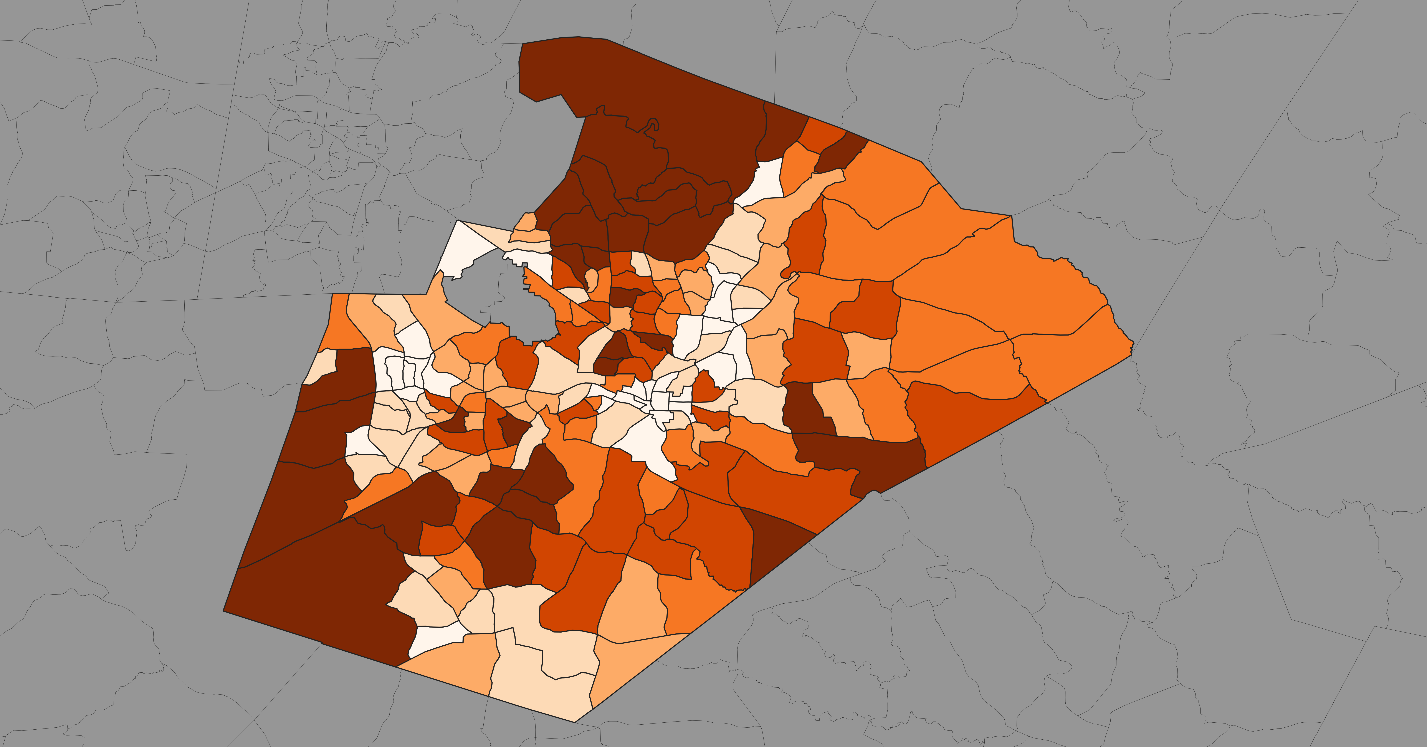


Average tree canopy percent

a


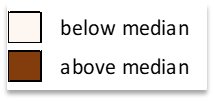

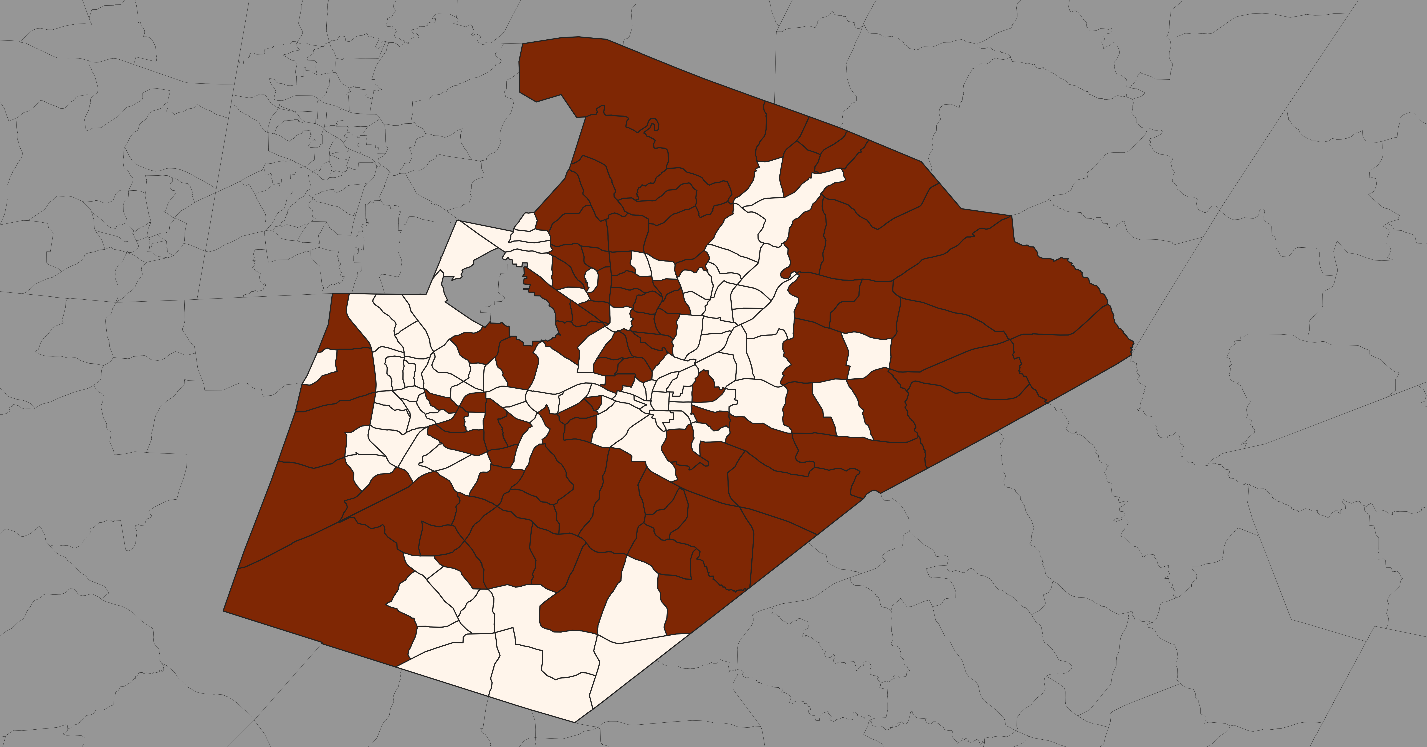


Average tree canopy

b


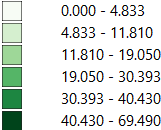
Supplemental Figure S.4: Distribution of forest land cover (percent) across Wake County, six evenly spaced categories (a) and above (green) and below median (b)
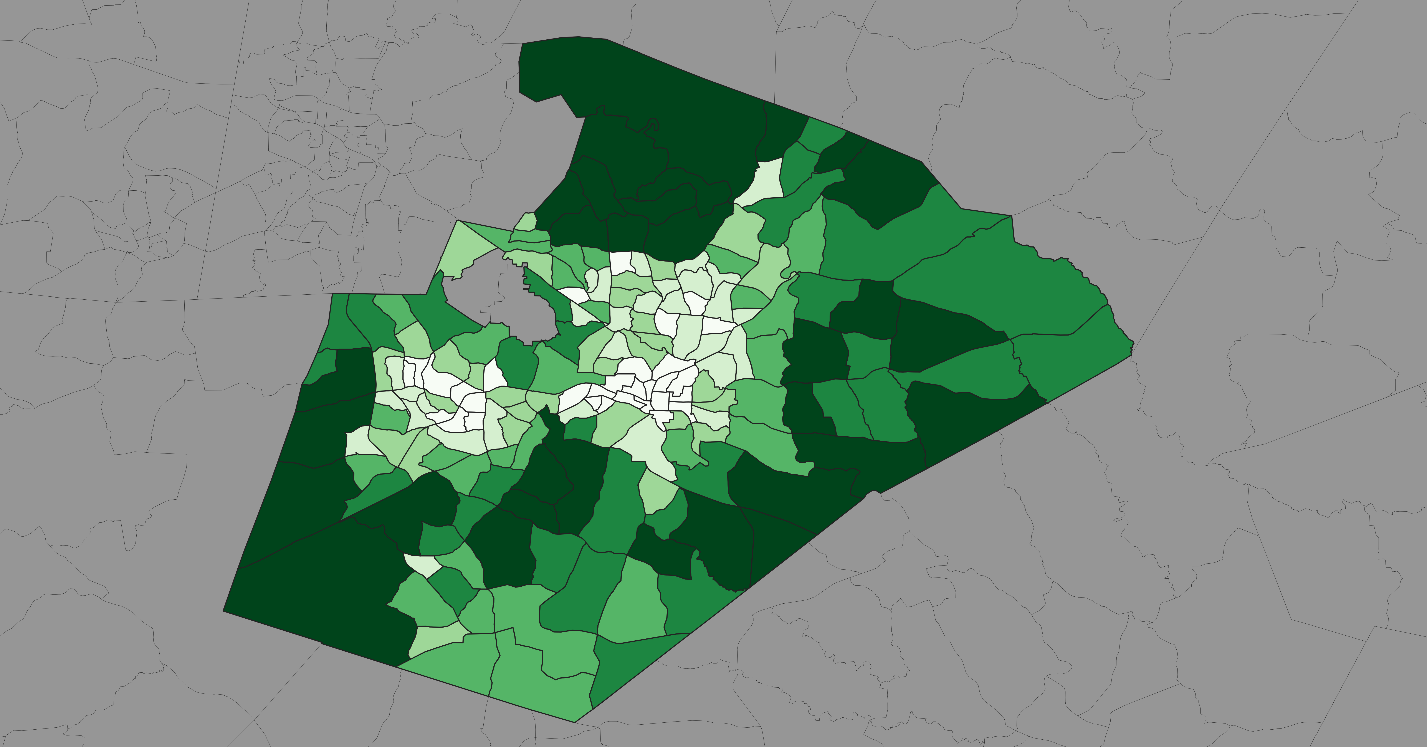


Forest land cover percent

a


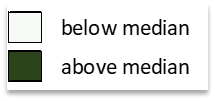

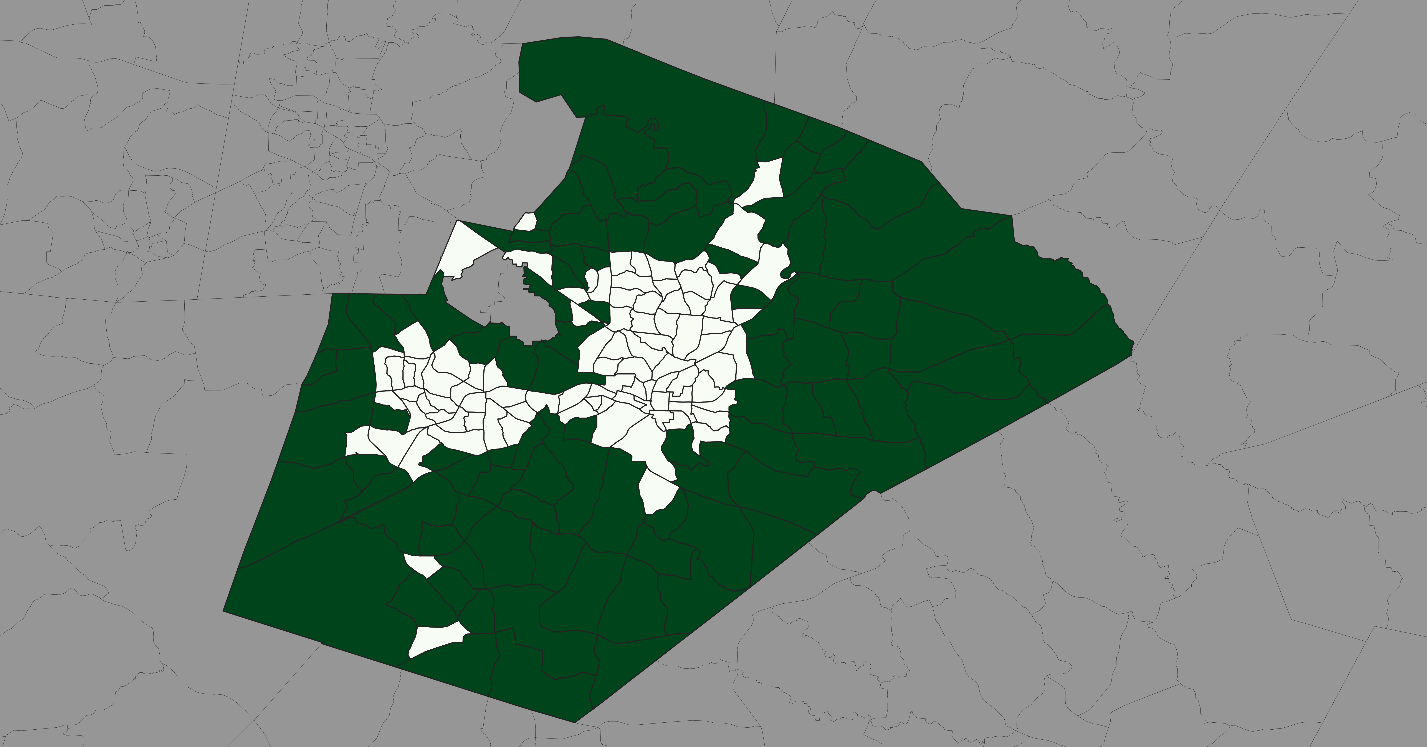


Forest land cover

b


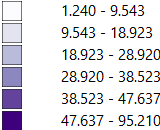
Supplemental Figure S.5: Distribution of urban land cover (percent) across Wake County, six evenly spaced categories (a) and above (purple) and below median (b)
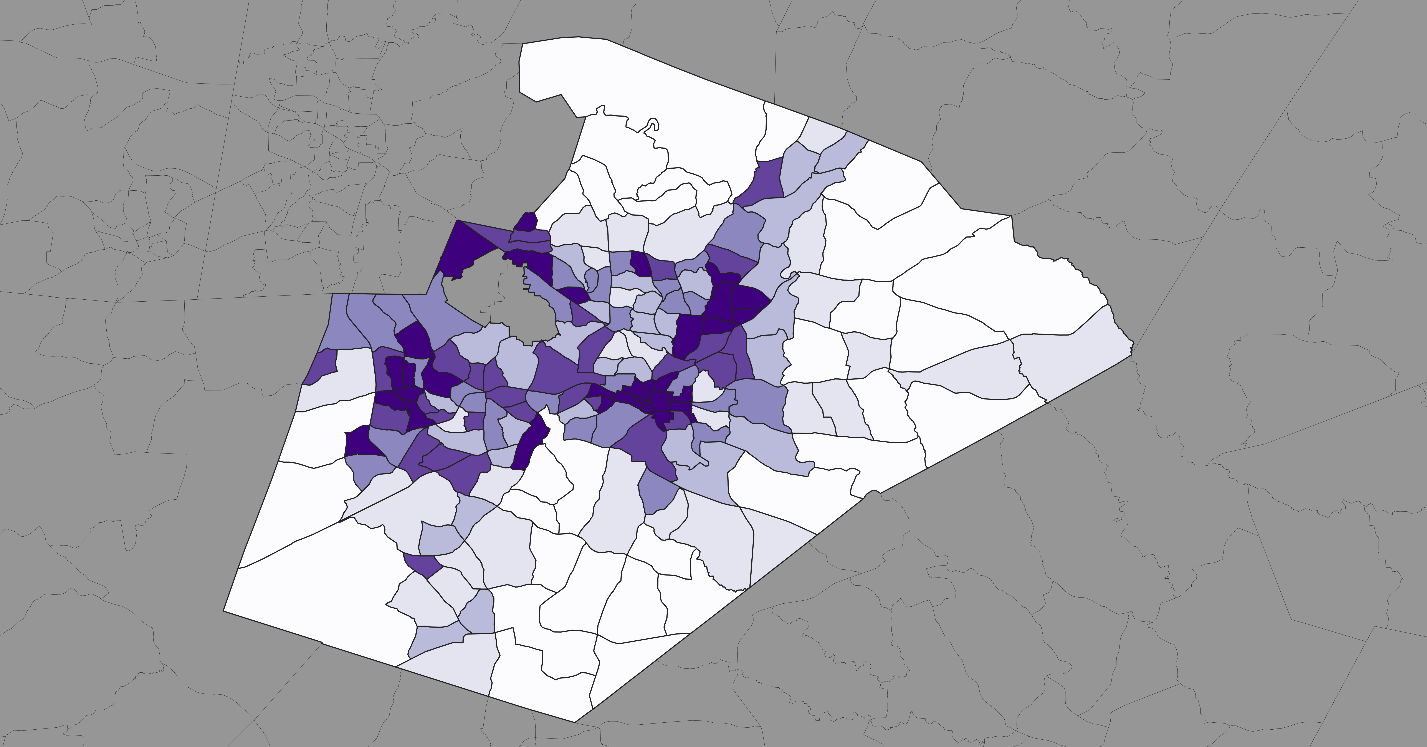


Urban land cover percent

a


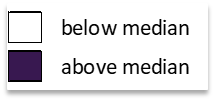

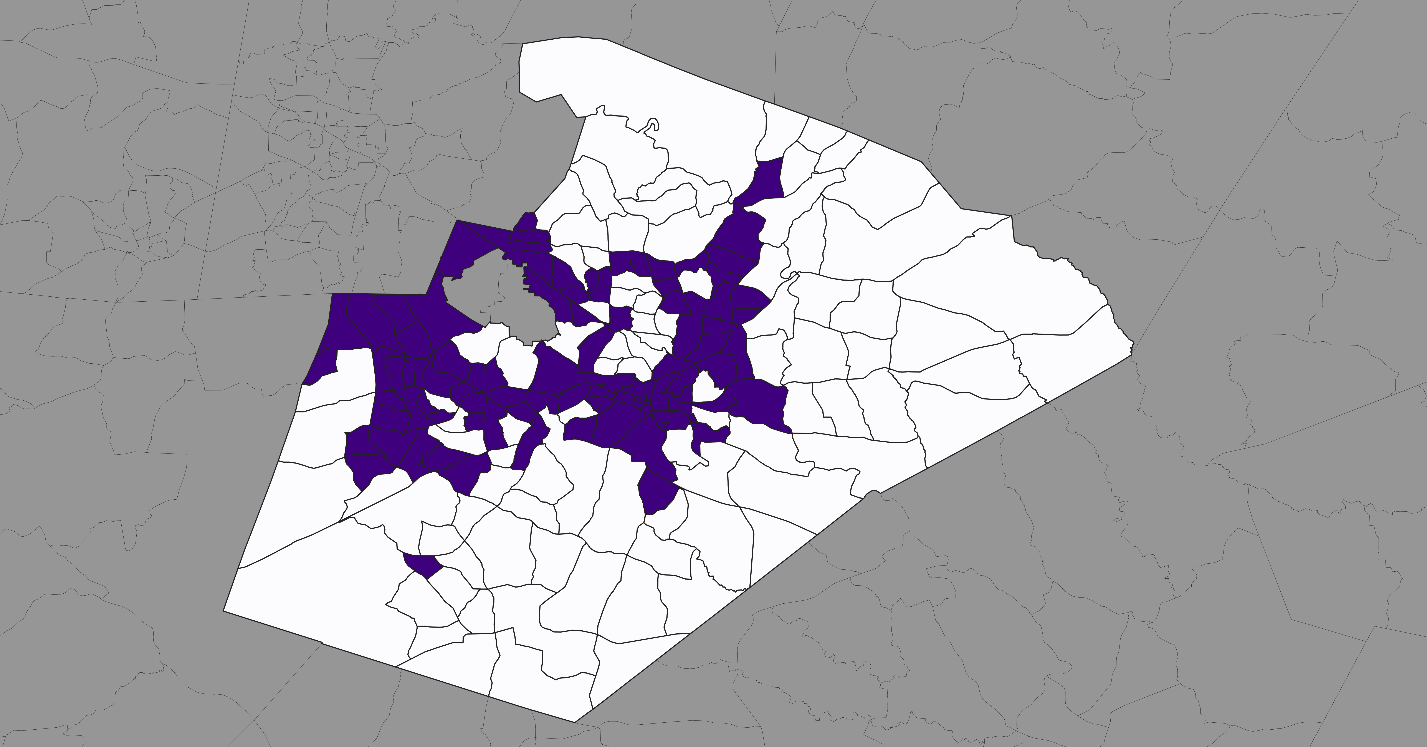


Urban land cover

b

## Supplemental Table S.1: Seasonal distributions of PM_2.5_, relative humidity, and temperature across the study period

| Season | Variable | Mean | SD | Min | 25th | 50th | 75th | Max | IQR |
| --- | --- | --- | --- | --- | --- | --- | --- | --- | --- |
| Spring | Daily average PM_2.5_ (µg/m^3^) | 10.5 | 4.3 | 4.7 | 8.42 | 10.5 | 14.14 | 31.14 | 5.73 |
|  | Relative humidity (%) | 59.02 | 16.19 | 20.63 | 46.42 | 58.21 | 69 | 95.88 | 22.58 |
|  | Temperature (oF) | 59.63 | 12.99 | 25.29 | 49.33 | 61.92 | 70.33 | 79.45 | 21 |
| Summer | Daily average PM_2.5_ (µg/m^3^) | 9.96 | 3.83 | 2.14 | 7.24 | 9.75 | 12.12 | 26.7 | 4.88 |
|  | Relative humidity (%) | 68.65 | 13 | 3.08 | 63.21 | 69.58 | 76.96 | 92.46 | 13.75 |
|  | Temperature (oF) | 76.68 | 4.02 | 66.41 | 74.25 | 76.83 | 79.54 | 85.67 | 5.29 |
| Fall | Daily average PM_2.5_ (µg/m^3^) | 10.04 | 4.15 | 1.82 | 7.42 | 9.25 | 12.25 | 21.08 | 4.83 |
|  | Relative humidity (%) | 70.57 | 12.41 | 32.88 | 63.42 | 70.63 | 79.42 | 93.46 | 16 |
|  | Temperature (oF) | 60.14 | 12.28 | 30.67 | 52.88 | 62.29 | 69.17 | 83.25 | 16.29 |
| Winter | Daily average PM_2.5_ (µg/m^3^) | 12.25 | 4.59 | 4.51 | 8.93 | 11.36 | 14.52 | 27.34 | 5.59 |
|  | Relative humidity (%) | 63.45 | 17.66 | 26.08 | 48.9 | 64.42 | 78.15 | 96 | 29.25 |
|  | Temperature (oF) | 40.79 | 10.26 | 14.54 | 35.08 | 40.6 | 46.98 | 70.79 | 11.9 |

## Supplemental Table S.2: Mortality odds ratios and 95% CIs for 5µg/m^3^ increase in PM_2.5_ 1 day before recorded sudden death stratified by individual and area level characteristics, for full population and subset population with diagnosed kidney and stroke patients removed.

| Characteristic | Level | Full population | | Subset population (stroke and chronic kidney disease patients removed) | |
| --- | --- | --- | --- | --- | --- |
|  |  | N | OR (95% CI) | N | OR (95% CI) |
| Unstratified |  | 399 | 1.18 (0.98, 1.41) | 337 | 1.35 (1.10, 1.65) |
| Left ventricular hypertrophy | Absent | 241 | 1.07 (0.85, 1.36) | 191 | 1.31 (1.00, 1.71) |
|  | Present | 62 | 1.90 (1.04, 3.50) | 51 | 2.80 (1.27, 6.19) |
| Neutrophil Lymphocyte Ratio | Absent | 55 | 0.83 (0.48, 1.42) | 44 | 1.02 (0.57, 1.85) |
|  | Present | 123 | 1.25 (0.89, 1.76) | 88 | 1.59 (1.04, 2.43) |
| BMI | <25 | 181 | 1.04 (0.79, 1.36) | 159 | 1.17 (0.87, 1.56) |
|  | >=25 | 218 | 1.28 (0.99, 1.66) | 178 | 1.54 (1.13, 2.10) |
| Greenway density | Above median | 204 | 1.00 (0.76, 1.31) | 177 | 1.20 (0.89, 1.63) |
|  | Below median | 192 | 1.38 (1.07, 1.79) | 158 | 1.60 (1.18, 2.17) |
| Forest land cover | Above median | 198 | 1.09 (0.84, 1.41) | 167 | 1.20 (0.90, 1.60) |
|  | Below median | 198 | 1.30 (0.99, 1.70) | 168 | 1.61 (1.18, 2.20) |
| Urban land | Above median | 198 | 1.15 (0.87, 1.51) | 163 | 1.55 (1.12, 2.14) |
|  | Below median | 198 | 1.18 (0.92, 1.52) | 172 | 1.23 (0.93, 1.64) |
| Average tree canopy | Above median | 196 | 1.07 (0.80, 1.43) | 172 | 1.33 (1.01, 1.75) |
|  | Below median | 200 | 1.23 (0.96, 1.58) | 163 | 1.38 (0.99, 1.94) |
| Census tract median income | Above median | 131 | 1.04 (0.74, 1.47) | 120 | 1.17 (0.82, 1.68) |
|  | Below median | 265 | 1.28 (1.03, 1.60) | 215 | 1.50 (1.16, 1.94) |
| Coronary artery disease | Absent | 278 | 1.28 (1.03, 1.58) | 242 | 1.49 (1.17, 1.90) |
|  | Present | 96 | 0.97 (0.63, 1.51) | 70 | 1.06 (0.59, 1.92) |
| Chronic respiratory disease | Absent | 248 | 1.20 (0.94, 1.52) | 216 | 1.33 (1.02, 1.74) |
|  | Present | 123 | 1.17 (0.83, 1.64) | 93 | 1.47 (0.96, 2.24) |
| Chronic kidney disease | Absent | 327 | 1.31 (1.06, 1.62) | - | - |
|  | Present | 44 | 0.58 (0.30, 1.11) | - | - |
| Diabetes | Absent | 263 | 1.20 (0.94, 1.54) | 241 | 1.31 (1.02, 1.70) |
|  | Present | 115 | 1.15 (0.83, 1.59) | 75 | 1.55 (0.97, 2.47) |
| Dyslipidemia | Absent | 227 | 1.47 (1.14, 1.88) | 212 | 1.51 (1.16, 1.98) |
|  | Present | 150 | 0.88 (0.65, 1.20) | 103 | 1.21 (0.81, 1.80) |
| Stroke | Absent | 345 | 1.24 (1.01, 1.51) | - | - |
|  | Present | 26 | 0.36 (0.11, 1.18) | - | - |
| Clinical condition* | None | 103 | 1.24 (0.85, 1.81) | 103 | 1.24 (0.85, 1.81) |
|  | One | 121 | 1.73 (1.15, 2.61) | 117 | 1.83 (1.20, 2.77) |
|  | More than one | 147 | 0.91 (0.67, 1.24) | 89 | 1.24 (0.82, 1.86) |
| *for full population includes: coronary artery disease, chronic respiratory disease, chronic kidney disease, diabetes, dyslipidemia, and stroke; for subset population coronary artery disease, chronic respiratory disease, diabetes, and dyslipidemia. Note that the dashed line indicates no estimation for those strata, as individuals with these conditions were removed for sensitivity analyses. | | | | | |


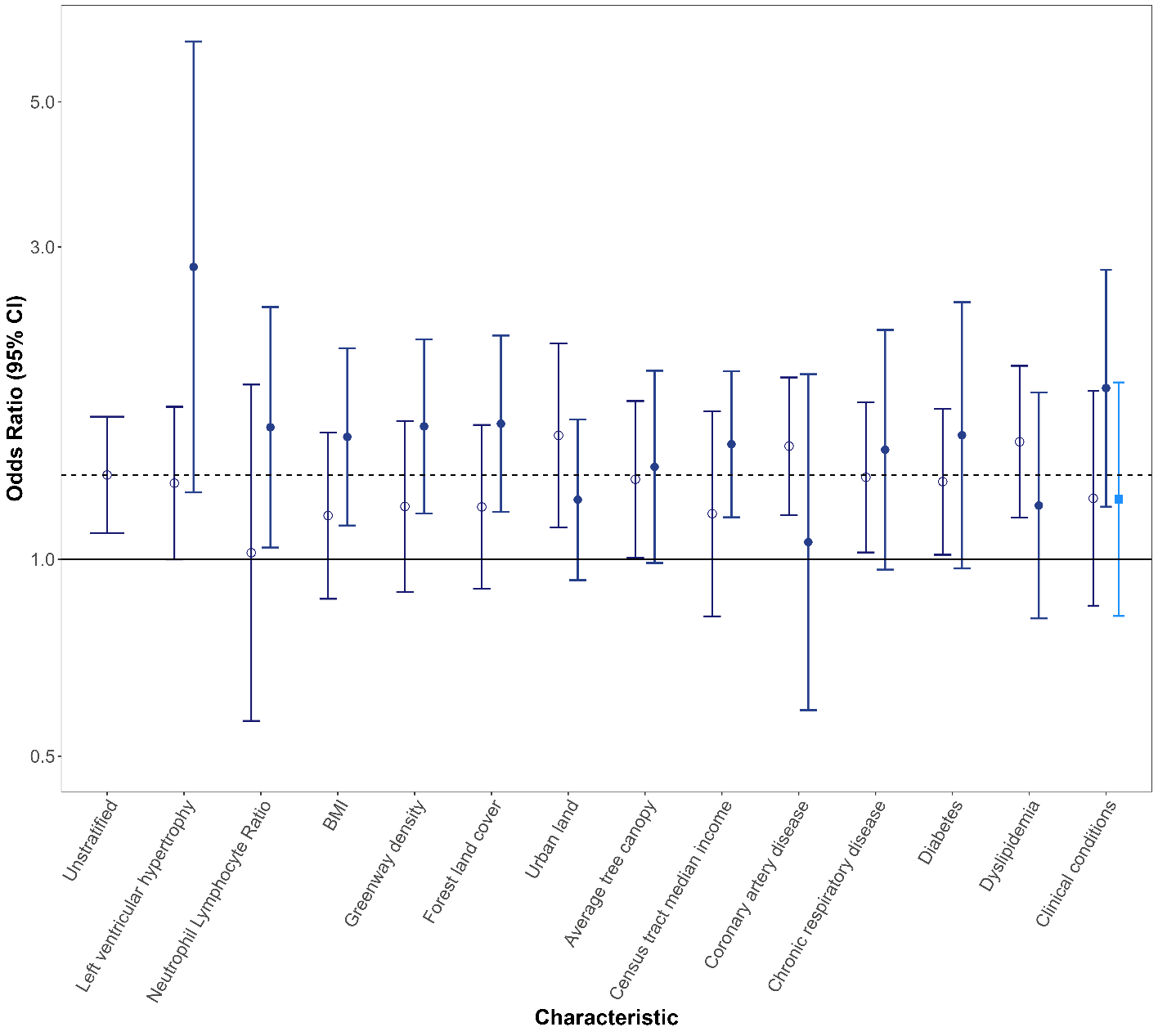


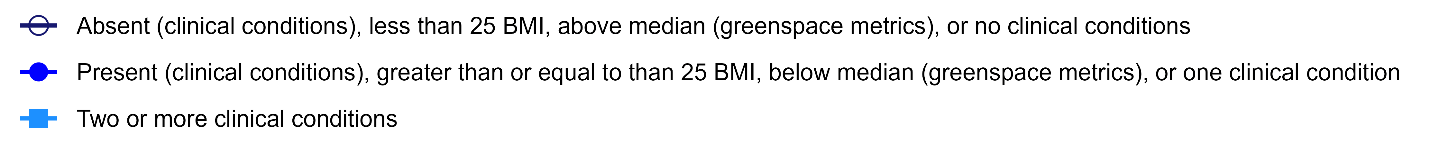


Supplemental Figure S.6: Mortality odds ratios and 95% CIs for 5µg/m^3^ increase in PM_2.5_ 1 day before recorded sudden death stratified by individual and area level characteristics, for subset population with patients with kidney and stroke disease removed. Darker blue open circle OR: absent (clinical and specific clinical conditions), less than 25 BMI, above median (greenspace metrics), or no clinical conditions. Medium blue closed circle OR: present (clinical and specific clinical conditions), greater than or equal to than 25 BMI, below median (greenspace metrics), or one clinical condition. Light blue square OR: two or more clinical conditions.
